# Psychometric properties and construct validity of the Parkinson’s Disease-Cognitive Rating Scale (PD-CRS) in Colombia

**DOI:** 10.3389/fpsyg.2022.1018176

**Published:** 2022-12-01

**Authors:** Hugo Juan Camilo Clavijo-Moran, Daniela Álvarez-García, Gabriel D. Pinilla-Monsalve, Beatriz Muñoz-Ospina, Jorge Orozco

**Affiliations:** ^1^Centro de Investigaciones Clínicas, Fundación Valle del Lili, Cali, Colombia; ^2^Departamento de Neurocirugía, Fundación Valle del Lili, Cali, Colombia; ^3^Laboratorio i2T/CENIT, Universidad Icesi, Cali, Colombia; ^4^Facultad de Ciencias de la Salud, Universidad Icesi, Cali, Colombia; ^5^Departamento de Neurología, Sección de Neuropsicología de Adultos, Fundación Valle del Lili, Cali, Colombia; ^6^Departamento de Neurología, Fundación Valle del Lili, Cali, Colombia

**Keywords:** Parkinson’s disease, PD-CRS, psychometric, cognitive impairment, Parkinson’s disease dementia

## Abstract

**Background:**

Cognitive impairment is frequent among people living with Parkinson’s disease: up to 40% of patients exhibit symptoms of mild cognitive impairment and 25% meet the criteria for dementia. Parkinson’s Disease Cognitive Rating Scale (PD-CRS) is one of the recommended scales by the Movement Disorders Society Task Force for level 1 screening of dementia. However, its psychometric properties have not been studied in the Colombian population.

**Methods:**

A cross-sectional study was conducted on 100 patients with Parkinson’s disease diagnosed by a movement disorders neurologist. Patients were evaluated with PD-CRS and MoCA. Principal component analysis was conducted, and then confirmatory factor analysis was implemented through the maximum-likelihood method. Internal consistency was evaluated using Cronbach *α*. Convergent and divergent validity were also calculated and concurrent validity with the MoCA was assessed.

**Results:**

62% were males. Their median age was 68 years (IQR 57–74) and the median disease duration was 4 years (IQR 2–9). 77% were classified in early stages (Hoehn and Yahr stage ≤ 2), while the MDS-UPDRS part III score was 25 (IQR 15.5–38). In the principal component factor analysis, the pattern matrix unveiled a mnesic and a non-mnesic domain. Confirmatory factor analysis showed similar explanatory capacity (*λ* ≥ 0.50) for items other than naming (*λ* = 0.34). Cronbach’s *α* for the full 9-items instrument was 0.74. MoCA and PD-CRS total scores were correlated (*ρ* = 0.71, *p* = 0.000). Assuming a cut-off score of 62 points, there is an agreement of 89% with the definition of dementia by MoCA for Colombia (*κ* = 0.59; *p* = 0.000).

**Conclusion:**

PD-CRS has acceptable psychometric properties for the Colombian population and has significant correlation and agreement with a validated scale (MoCA).

## Introduction

Parkinson’s disease (PD) is the second most common neurodegenerative disease worldwide with an alarming growth rate (Dorsey et al., 2018; Dorsey and Bloem, 2018). Diagnosis of PD is based on parkinsonian motor symptoms such as bradykinesia, rigidity, rest tremor, and postural instability (Postuma et al., 2015). However, there is a high burden of non-motor symptoms throughout the course of the disease (Zis et al., 2015; Hermanowicz et al., 2019; Fernandes et al., 2021), even many years before the diagnosis (Fereshtehnejad et al., 2019; Heinzel et al., 2019). Cognitive impairment (CI) is a non-motor symptom among people living with PD that can arise prior to the motor symptoms onset (Darweesh et al., 2017; Fengler et al., 2017; Fereshtehnejad et al., 2019), at the moment of diagnosis or a few years after the disease onset (Aarsland et al., 2021). CI in Parkinson’s disease is frequent all along the course of the disease: mild cognitive impairment (MCI) prevalence in PD is 40% (Baiano et al., 2020), and it can be present in 20.2% at diagnosis (Pedersen et al., 2017). Furthermore, Parkinson’s disease dementia (PD-D) prevalence is between 25% and 30% rising to 83% at 20 years since diagnosis (Hely et al., 2008).

The cognitive profile in PD has a broad clinical spectrum. In order to explain this cognitive profile heterogeneity, the “Dual-Syndrome Hypothesis” has been proposed which states that patients with more fronto-striatal dysfunction have more attention, working memory, and executive functions compromise, whereas those with greater memory, language, and visuospatial compromise have more posterior cortical degeneration (Kehagia et al., 2013). Moreover, in early stages, there is usually a single non-amnesic impairment or dysexecutive syndrome, with relative preservation of core language features; nevertheless, the more sensitive predictors of progression to dementia are language and visuospatial compromise. However, every domain can be compromised, and some patients suffer from multidomain cognitive impairment (Aarsland et al., 2010; Litvan et al., 2011; Gonzalez-Latapi et al., 2021). Therefore, a comprehensive approach is needed for PD cognitive evaluation.

In order to standardize the diagnosis of cognitive impairment in Parkinson’s disease, expert panels have defined criteria for mild cognitive impairment (MCI) and dementia (Emre et al., 2007; Litvan et al., 2012). MCI diagnosis criteria include *“(1) having a diagnosis of Parkinson’s disease, (2) gradual cognitive decline reported by the patient, informant, or clinician, (3) cognitive decline based on a neuropsychological evaluation or using a global cognition scale validated in PD, and (4) cognitive decline that is not sufficient to interfere significantly with functional independence”* (Litvan et al., 2012). Parkinson’s disease dementia (PD-D) diagnosis is established based on *“(1) having the diagnosis of Parkinson’s disease, (2) cognitive impairment with an insidious onset and slow progression diagnosed by history, clinical and mental examination with impairment of more than one cognitive domain representing a change from premorbid level, (3) deficit severe enough to impair daily life, (4) associated clinical features (cognitive typical profile and/or behavioral symptoms), and (5) absence of features that make PD-D improbable such as the presence of other abnormalities that contribute to the dementia syndrome, probable vascular dementia diagnosis and presence of symptoms only in acute disease context or severe depression”* (Emre et al., 2007).

Current recommendations of the Movement Disorders Society Trask Force for assessing objective cognitive decline in PD include level 1 criteria for screening with recommended scales (Skorvanek et al., 2018) such as Montreal Cognitive Assessment (MoCA; Nasreddine and Phillips, 2005), Mattis Dementia Rating Scale on its second edition (MDRS-2; Griffiths et al., 2011), or the Parkinson’s Disease Cognitive Rating Scale (PD-CRS; Pagonabarraga et al., 2008); level 2 criteria consider neuropsychological evaluation where every cognitive domain has to be tested with at least two instruments (Litvan et al., 2012). Although it could be recommended to perform a full neuropsychological evaluation of cognition, it is not always possible in the clinical context. In consequence, level 1 scales, such as PD-CRS become relevant in daily practice. PD-CRS is an instrument that was specifically designed for PD cognitive evaluation and assesses cortical and subcortical functions (Pagonabarraga et al., 2008) which have been widely studied in diverse populations (Pagonabarraga et al., 2008; Martínez Martín et al., 2009; Fernández de Bobadilla et al., 2013; Santangelo et al., 2014; Fernández-Bobadilla et al., 2017; Samat et al., 2017; Serrano-Dueñas et al., 2017; Koevoets et al., 2018; Tan et al., 2020; Mahmoudi Asl et al., 2022); unfortunately, studies assessing this instrument’s consistency are heterogeneous in terms of patients’ clinical and sociodemographic characteristics (Rosca and Simu, 2020) and only one study has been carried out on Latin American population (Serrano-Dueñas et al., 2017).

The aim of this study was to evaluate the psychometric properties of the PD-CRS in a Colombian PD population and evaluate the concurrent validity with a level 1-recommended scale previously validated in the country (MoCA).

## Materials and methods

### Study design

A cross-sectional psychometric study was conducted between July 2018 and August 2021.

### Sample size

Considering the minimum necessary sample for conducting factor analysis recommended by Mundfrom et al. (2005), we defined a sample size of at least 90 subjects for achieving a good level criterion (*K* = 0.92) in the settings of an instrument (PD-CRS) with a two-factor solution (F2), a ratio of variables to factors (*p*/*f*) of 4.5, and a wide level of communality (0.2–0.8; Mundfrom et al., 2005).

### Instruments

MoCA (Nasreddine and Phillips, 2005) is a short cognitive screening tool that can be applied in 10 min and evaluates 7 cognitive domains: executive/visuospatial function, nomination, attention, language, abstraction, memory, and orientation. It is able to discriminate NC from MCI with a suggested cut-off point in the original validation study of 26, yielding 90% sensibility and 83% specificity; a suggested cut-off point of 18 is suggested to discriminate NC from dementia (Nasreddine and Phillips, 2005). MoCA has been validated to several languages including Colombian Spanish (Gil et al., 2015) with a global cut-off point of less than 22 suggesting MCI and less than 18 suggesting dementia (Pedraza et al., 2017). However, cut-off points vary depending on level of education.

PD-CRS is a cognitive screening tool specifically designed for Parkinson’s disease cognitive evaluation. The scale is divided into two sections: cortical and subcortical items based on neural correlates with clinical and imaging studies (Pagonabarraga et al., 2008). The subcortical section is composed of 10 items including attention; working memory; phonemic, semantic, alternating, and action verbal fluency; immediate and delayed verbal memory; and clock drawing. The cortical section contains two items: clock copy and naming (Pagonabarraga et al., 2008). This scale is able to discriminate NC from MCI and PD-D (Pagonabarraga et al., 2008). The original study suggested a cut-off point of 64 or less to differentiate NC from PD-D with a sensitivity and specificity of 94%. Subsequent studies have suggested 81 points or less as the cut-off point to differentiate NC from MCI with a sensitivity of 79% and a specificity of 80% (Pagonabarraga et al., 2008; Fernández de Bobadilla et al., 2013).

### Subjects and evaluation

Patients were non-randomly selected among those who attended a subspecialized neurology consultation at the Fundación Valle del Lili University Hospital (Cali, Colombia). We included patients that fulfilled the UK Parkinson’s Disease Society Brain Bank diagnostic criteria (Gibb and Lees, 1988). Patients with a doubtful diagnosis or suspected atypical parkinsonism, those with known major depressive disorder, coexistence with Alzheimer’s disease, or vascular dementia were excluded.

Patients were evaluated by a movement disorder specialist in order to confirm the diagnosis. Motor status and severity of the disease were determined using the Movement Disorders Society-sponsored revision of the Unified Parkinson’s Disease Ratings Scale (MDS-UPDRS) part III (Goetz et al., 2008) and the Hoehn and Yahr stages. Motor subtype was calculated as proposed by Stebbins et al. (2013); however, only items from the MDS-UPDRS III were considered. MoCA and PD-CRS were applied by a neuropsychologist on the same day fulfilling MDS level I criteria for PD-D.

### Statistical methods

Numeric variables were described with means (standard deviation) and/or medians (interquartile range) according to their distribution while categorical features were presented with absolute and relative frequencies. Data distribution was studied by analyzing PD-CRS score normality using the Kolmogorov–Smirnov test. The difference between the mean and median of the total score was expected to be less than 10% of the maximum observed. Floor and ceiling effects were considered significant if >15%. Hoehn and Yahr stage is a risk factor for cognitive impairment (Aarsland et al., 2021) and a ceiling effect related to early stages was expected; hence, the relationship between disease staging and total scores was analyzed using a multivariate robust linear regression with Huber/biweight iterations. Correlations were assessed using Pearson’s coefficient. Differences among known groups were determined with *t*-tests and one-way ANOVA with the Bonferroni *post-hoc* method.

Suitability for principal component factors analysis was identified using the Kaiser–Meyer–Olkin measure for sampling adequacy and the Bartlett test of sphericity. Relevant factors were extracted if their eigenvalues were > 1 and then the loadings were rotated using an orthogonal varimax without Kaiser. Loadings (*λ*) supported the relationship between an item and its factor if ≥0.4 with inter-factor differences ≥0.2. One dimensionality of retained factors was subsequently confirmed using the same procedure. Additionally, confirmatory factor analysis of exploratively obtained factor structure (van Prooijen and van der Kloot, 2001) through the maximum-likelihood method was implemented and goodness of fit was dependent on the coefficient of determination (>0.90), comparative fit index (>0.90), Tucker–Lewis index (>0.90), model vs. saturated *χ*^2^ (*p* > 0.050), root mean square error of approximation (<0.08), and standardized root mean square residual (<0.08). Invariance by motor subtype and global cognitive function was studied.

Cronbach’s *α* was calculated as a measure of internal consistency for total and subtotal scores. The two-halve procedure was included with the same purpose. Minimum inter-test/rest correlation and changes in Cronbach’s *α* after item removal were evaluated. Concurrent validity with the total score of MoCA was estimated using an intraclass correlation coefficient (ICC) and interpreted from Pearson’s correlation coefficients when studying the subtotal scores for the PD-CRS retained factors in relation to MoCA subtests. Total PD-CRS scores were described in comparison to MoCA limit scores for minimal cognitive impairment and dementia in the Colombian population (Pedraza et al., 2017). We calculated the chance-corrected agreement (*κ*) between MoCA and PD-CRS for the dementia category using the cut-off proposed by Serrano-Dueñas et al. (2017) in a neighboring population (62 points).

Significant *p* values were considered if <0.050. Analyses were performed in Stata v.16. (StataCorp, Texas, USA).

## Results

### Sample description

One hundred Parkinson’s disease patients were included in the study, 62% were males. The median age was 68 years (IQR 57–74). PD diagnosis was assigned at a mean age of 59.49 ± 11.58 years, representing a median disease length of 4 years (IQR 2–9). 45% was ranked in stage 2 on the Hoehn & Yahr (H&Y) Scale. The median MDS-UPDRS score was 25 (IQR 15.5–38) and 84% were classified in the postural instability and gait disorder (PIGD) subtype (Table 1).

**Table 1:** Sociodemographic and clinical characteristics of the sample.

Variable	*n*:100
Age	68 (IQR 57–64)
Female sex	38%
Education (≤ 11 years)	37%
Age at PD diagnosis	59.49 ± 11.58
Disease duration (years)	4 (IQR 2–9)
Hoehn & Yahr stage	
1	16%
1.5	16%
2	45%
2.5	8%
3	12%
4	3%
MDS-UPDRS III	25 (IQR 15.5–38)
Motor subtype	
PIGD	84%
TD	14%
Undetermined	2%
PD-CRS score	
Total	78.32 ± 18.64
Cortical	26.08 ± 5.07
Subcortical	52.24 ± 16.07
MoCA score	22.6 ± 4.16

### Data acceptability

Information was fully computable and there were no missing data. Total scores of the studied instrument showed a normal distribution according to the Kolmogorov–Smirnov test (*D* = 0.09, *p* = 0.691). The average score was 78.32 points with a standard deviation of 18.64 and a standard error of 1.86; the median score was 80 with an interquartile range of 23. Minimum and maximum scores were 31 and 117, respectively. The difference between the mean and the median was −1.43% of the maximum score. As the mean is less than the median, skewness was negative at −0.41; additionally, there was a tendency toward a light-tailed distribution as kurtosis was 2.82.

### Description of known-group scores

The total score was significantly and inversely correlated with age (Pearson’s *ρ* = −0.40, *p* = 0.000) and years of disease (Pearson’s *ρ* = −0.26, *p* = 0.009). There were no significant differences according to sex (*t* = −0.53, dof 98, *p* = 0.597), but scores were higher for those with more than 12 years of education (83.92 ± 15.73 vs. 68.78 ± 19.51; *t* = 4.24, *p* < 0.001).

**Figure 1:**
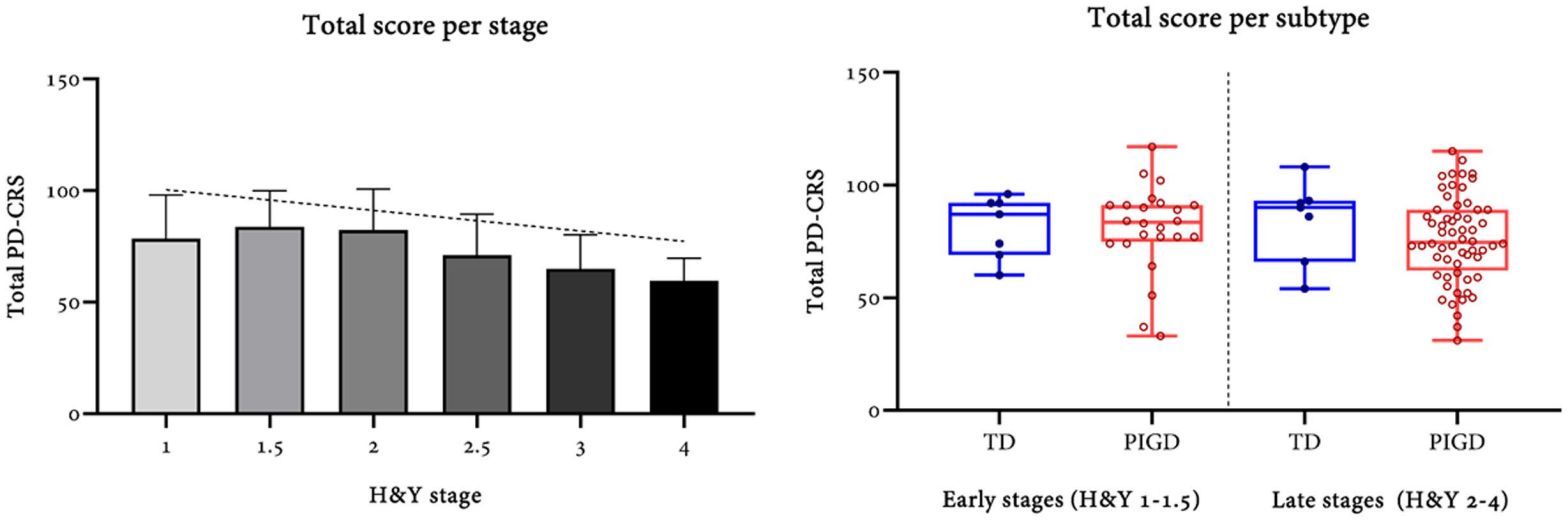
Total PD-CRS per MDS motor subtype and stage of disease according to the Hoehn and Yahr classification.

PD-CRS score exhibited global significant differences (*p* = 0.014) according to Hoehn and Yahr stages, as follows: 1 (78.5 ± 19.57, *n* = 16), 1.5 (83.75 ± 16.13, *n* = 16), 2 (82.38 ± 18.36, *n* = 45), 2.5 (71.13 ± 18.45, *n* = 8), 3 (65.08 ± 15.28, *n* = 12), and 4 (59.67 ± 10.02, *n* = 3). A significant correlation coefficient (Pearson’s *ρ* = −0.275, *p* = 0.006) between stage and the total score was also found (Figure 1). On the contrary, there was no important relation with the MDS-UPDRS score (Pearson’s *ρ* = −0.19, *p* = 0.053).

No differences were found between the tremorous (82.78 ± 15.55, *n* = 14), postural instability-gait disorder (77.29 ± 19.9, *n* = 84), and indeterminate (90.50 ± 0.71, *n* = 2) subtypes (*F* = 0.96, dof = 97, *p* = 0.388). Furthermore, no significant differences were found in the subcortical (*p* = 0.532) and cortical (*p* = 0.185) scores by motor subtype.

Regarding the distribution of scores on each test, there was an important ceiling effect for naming, sustained attention, clock drawing, clock copy, and the cortical subtotal score. The sustained attention test was the only one demonstrating a floor effect >15% (Table 2).

**Table 2:** Acceptability of the PD-CRS tests, sub-scores, and total score.

Variable	Mean	Median	Mean-Med	10% Max	Floor (%)	Ceiling (%)
Immediate verbal memory	7.05	7	0.05	1.20	1	1
Naming	16.94	19	−2.06	2.00	3	31
Sustained attention	6.37	8	−1.63	1.00	16	22
Working memory	2.93	3	−0.07	0.90	14	0
Clock drawing	8.28	9	−0.72	1.00	2	31
Clock copy	9.14	9.5	−0.36	1.00	0	50
Delayed verbal memory	4.13	4	0.13	1.00	10	0
Alternating fluency	10.12	10	0.12	2.00	0	4
Action fluency	13.36	13	0.36	3.00	0	3
Cortical score	26.08	28	−1.92	3.00	0	19
Subcortical score	52.24	53.5	−1.26	8.80	0	0
Total score	78.32	80	−1.68	11.70	0	0

A multivariate analysis using robust linear regression demonstrated that the relationship between Hoehn and Yahr stage and total PD-CRS score remains significant at *p* = 0.012 (coefficient − 3.23 95% CI: –5.72 to –0.74, SE = 1.26, *t* = 2.57) after adjusting by age (*p* = 0.0000) and < 12 years of education (*p* = 0.000).

### Construct validity

Conditions for factor analysis were confirmed with a Kaiser–Meyer–Olkin measure of 0.77 for sampling adequacy and a Bartlett test of sphericity (*χ*^2^ = 253.14, dof = 36, *p* = 0.000) that allowed the rejection of the null hypothesis suggesting lack of intercorrelation between variables. Nonetheless, the determinant of the correlation matrix was borderline at 0.07. Principal component factors analysis exhibited two retained factors with a proportion of 0.40 attributed to the first dimension and 0.13 for the second, with a cumulative proportion of 0.53. Factor loadings were then evaluated using an orthogonal varimax without Kaiser rotation.

The pattern matrix showed two different factors: a non-mnesic dimension (action fluency, alternating fluency, clock drawing, working memory, clock copy, sustained attention, and naming) and an mnesic domain (delayed and immediate verbal memory) (Table 3). Differences between loading for each factor were ≥ 0.2, except for sustained attention. After the rotation, the proportion attributed to non-mnesic and mnesic factors were 0.31 and 0.22, in the same order. One-dimensionalities were confirmed (explained proportions of variance were 0.44 and 0.82 with one factor).

**Table 3:** Factor loadings for non-mesic and mnesic domains of PD-CRS in Colombia.

Variable	Non-mnesic	Mnesic	Difference	Uniqueness	Commonality
Action fluency	0.751	0.027	0.724	0.436	0.564
Alternating fluency	0.730	0.195	0.535	0.429	0.571
Clock drawing	0.655	0.270	0.385	0.498	0.502
Working memory	0.619	0.341	0.278	0.499	0.501
Clock copy	0.598	0.189	0.409	0.610	0.390
Sustained attention	0.557	0.403	0.154	0.527	0.473
Naming	0.441	0.091	0.350	0.797	0.203
Delayed verbal memory	0.166	0.878	0.712	0.202	0.798
Immediate verbal memory	0.093	0.876	0.783	0.224	0.776

**Figure 2:**
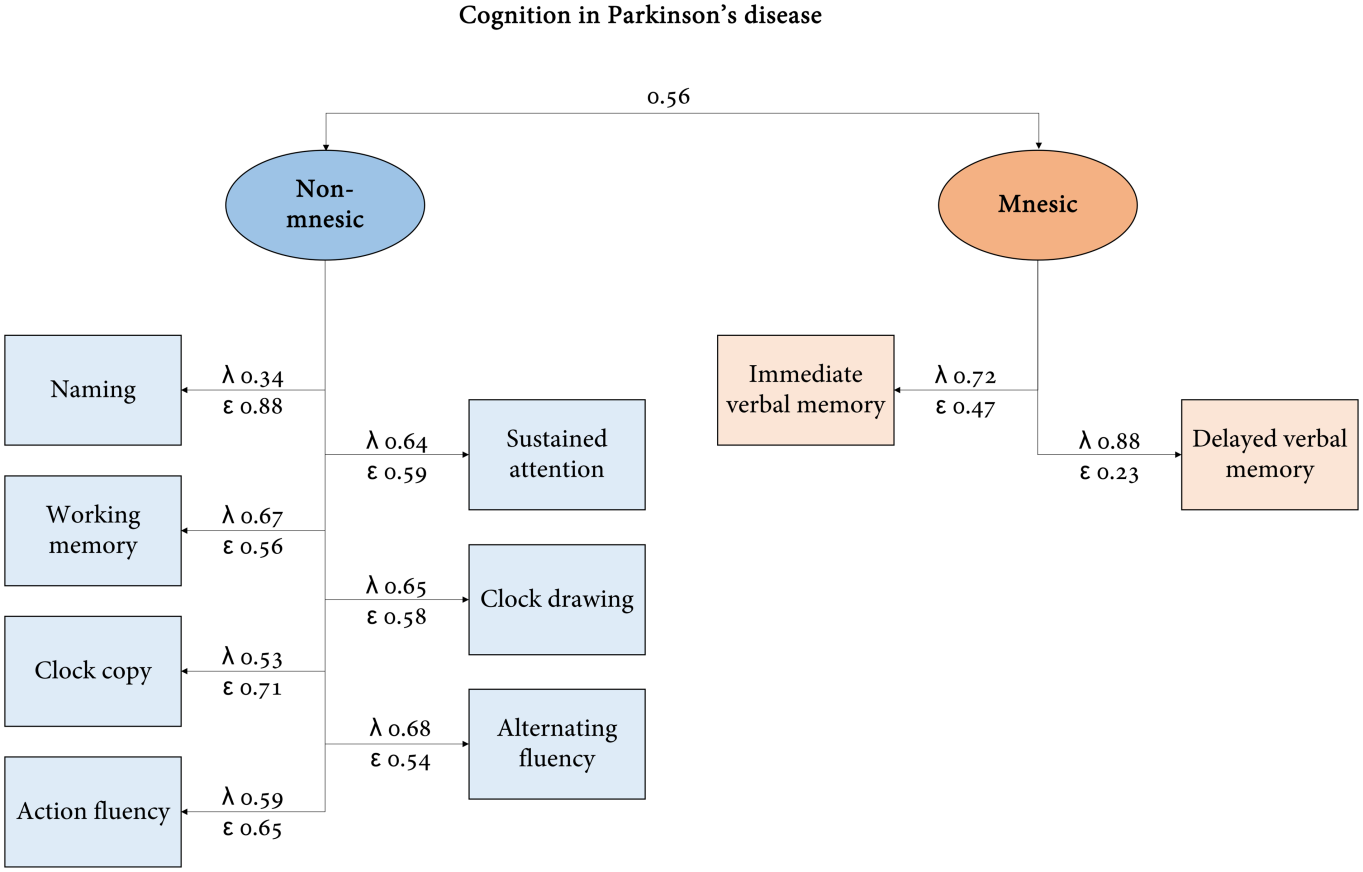
Structural model for cognition in PD by domains and tests.

Confirmatory factor analysis (Figure 2) showed covariance between both dimensions (*p* = 0.000). Similar explanatory capacity (*λ* ≥ 0.50) in the non-mnesic domain was detected for items other than naming (*λ* = 0.34) and variance explanation was higher for delayed verbal memory (*λ* = 0.88) in the mnesic factor. Regarding the goodness of fit, satisfactory results were obtained for the coefficient of determination (0.96), comparative fit index (0.95), Tucker–Lewis index (0.93), and *χ*^2^ likelihood ratio (model vs. saturated, *χ*^2^ = 37.14, *p* = 0.073). RMSEA and SRMR were acceptable but not optimal at 0.06 and 0.05, respectively.

There was support for metric and scalar invariances [*p* > 0.050, (∆*χ*^2^/∆dof) < 3] but not for strict invariance when comparing PIGD vs. tremorous/undetermined subtypes and MCI/dementia vs. apparently normal cognition by MoCA (see Supplementary material).

If the original structure of the PD-CRS is presumed (cortical and subcortical dimensions), the goodness of fit is numerically lower: CD (0.84), CFI (0.81), TLI (0.74), *χ*^2^ likelihood ratio (*χ*^2^ = 69.40, *p* = 0.000), RMSEA (0.13), and SRMR (0.08).

### Convergent and divergent validity

There was a positive but weak correlation between the original subcortical and cortical scores (Pearson’s *ρ* = 0.39, *p* < 0.001). Likewise, there was a significant positive correlation between the proposed non-mnesic and mnesic scores (Pearson’s *ρ* = 0.42, *p* = 0.000).

Correlation coefficients among items within the non-mnesic dimension range from 0.09 (naming-sustained attention) to 0.59 (action-alternating fluencies), with a median of 0.34. Immediate and delayed verbal memories (mnesic factor) were significantly correlated (Pearson’s *ρ* = 0.64, *p* = 0.000) and this coefficient was higher in comparison to any other inter-factor correlation with the non-mnesic items.

### Internal consistency

Internal consistency (Cronbach’s *α*) for the full 9-item instrument was 0.74 but reached only 0.37 when using the subcortical and cortical subtotal scores. Similarly, there was an acceptable consistency for the subcortical factor (7 items, *α* = 0.71), but it was less than poor for the cortical dimension (2 items, *α* = 0.20). In the two-half procedures, items were randomly categorized into two groups (g1 with 5 and g2 with 4 items), showing a significant positive correlation among them (Pearson’s *ρ* = 0.57, *p* = 0.000), with an acceptable consistency for both groups (*α*1 = 0.67 and *α*2 = 0.68).

The minimum item-test correlation was 0.51 for immediate verbal memory and the minimum item-rest correlation was 0.30 for naming. Cronbach’s *α* after removal of each item was lower than that of the 9-item scale, except for naming (*α* = 0.75), representing an increase in consistency of only 0.004. On the other hand, Cronbach’s *α* is reduced to 0.68 if the alternating fluency item is removed. Therefore, all nine tests were considered relevant to the final score.

For the proposed non-mnesic (*α* = 0.71) and mnesic factors (*α* = 0.76), internal consistency was also acceptable.

### Concurrent validity

**Figure 3:**
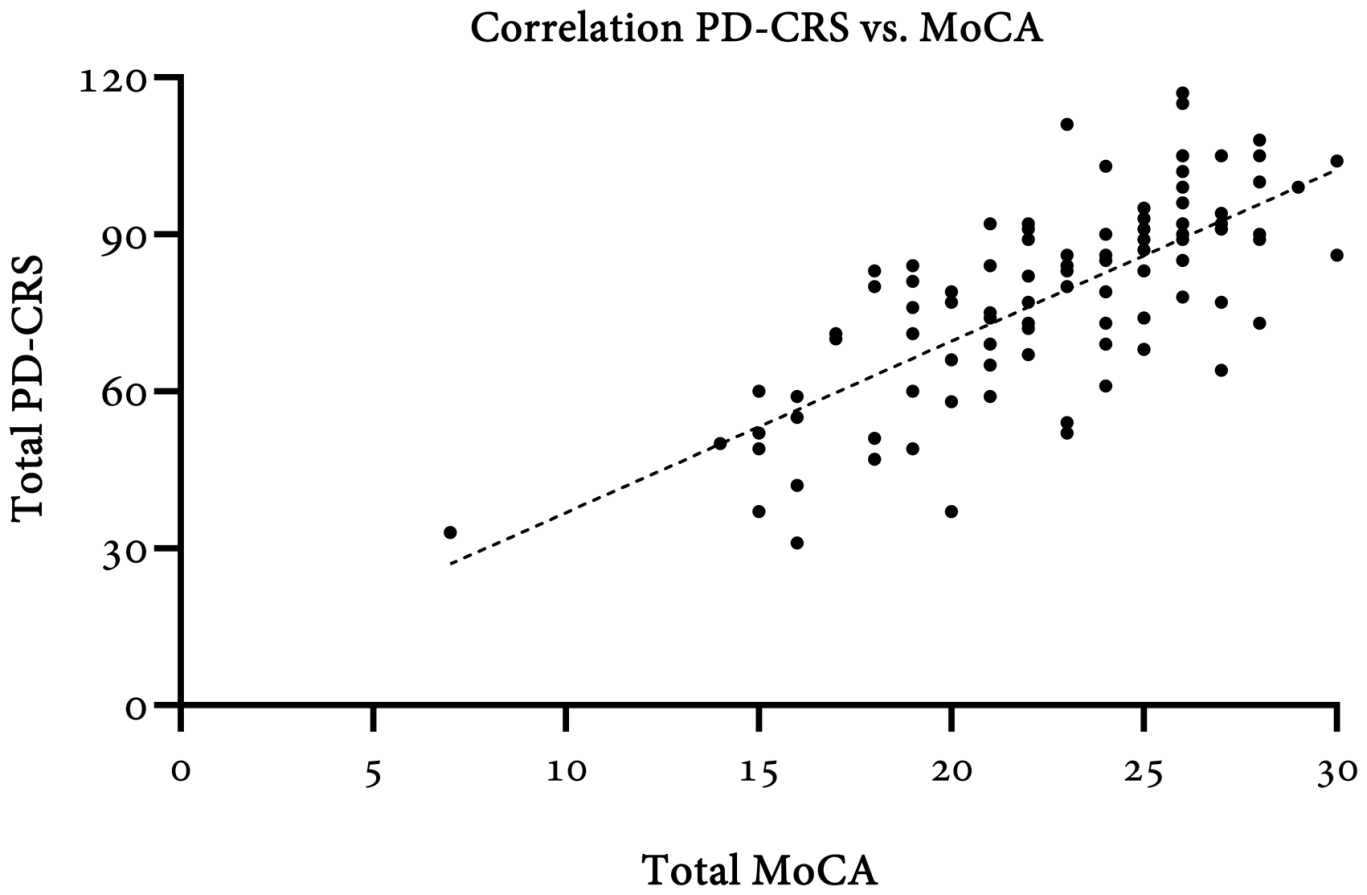
Correlation between MoCA and PD-CRS total scores.

Concurrency was assessed in comparison to the MoCA test (Table 4). Total scores showed moderate agreement (ICC = 0.47; 95%CI: 0.22–0.65) and a strong correlation (Pearson’s *ρ* = 0.71, *p* = 0.000) (Figure 3). MoCA subtests were significantly correlated with PD-CRS total and non-mnesic domain scores. Visuospatial (Pearson’s *ρ* = 0.19, *p* = 0.059), attention (Pearson’s *ρ* = 0.19, *p* = 0.060), language (Pearson’s *ρ* = 0.14, *p* = 0.154), and abstraction (Pearson’s *ρ* = 0.04, *p* = 0.687) subtests were not significantly associated when compared with the mnesic domain. Nonetheless, the MoCA delayed recall score was correlated with total and subtotal PD-CRS scores.

**Table 4:** Correlations for total and subtotal scores of MoCA with PD-CRS.

MoCA	PD-CRS					
	Non-mnesic		Mnesic		Total	
	*p*	Value of p	*p*	Value of p	*p*	Value of p
Visuospatial	0.557	0.000	0.190	0.059	0.536	0.000
Naming	0.263	0.008	0.232	0.020	0.284	0.004
Attention	0.507	0.000	0.189	0.060	0.492	0.000
Language	0.424	0.000	0.144	0.154	0.409	0.000
Abstraction	0.32	0.001	0.041	0.687	0.298	0.003
Delayed recall	0.435	0.000	0.515	0.000	0.499	0.000
Orientation	0.353	0.000	0.358	0.000	0.391	0.000
Total	0.710	0.000	0.457	0.000	0.731	0.000

Taking into account the MoCA cut-off values for the Colombian population (Pedraza et al., 2017), patients with normal cognition (MoCA ≥ 23) had a PD-CRS of 87.23 ± 14.25, while those with mild cognitive impairment (MoCA ≤ 22) and dementia (MoCA≤17) scored 71.57 ± 13.85 and 48.90 ± 12.17, respectively. Significant differences were found among the three groups (*p* = 0.000), and even between the MCI and dementia groups (*p* = 0.000). Assuming an optimal cut-off of 62 points for the total PD-CRS as proposed by Serrano-Dueñas et al. (Serrano-Dueñas et al., 2017) in a neighboring population, there is a significant agreement of 89% with the definition of dementia by MoCA (κ = 0.59, *p* = 0.000). Area under the ROC curve of total PD-CRS against MoCA for PD-D was 0.95.

## Discussion

This study shows that PD-CRS is a reliable, acceptable, and useful instrument for evaluation of PD patients in the Colombian population. Factorial analysis suggests a different grouping for sub-items of the scales into non-mnesic and mnesic domains. Internal consistency is acceptable for all 9 items (*α* = 0.74) as well as for amnesic (*α* = 0.76) and non-amnesic dimensions (*α* = 0.71). PD-CRS shows a significant correlation with MoCA as well as statistically different scores in the normal cognition (NC), MCI, and dementia groups based on MoCA. Good agreement was found between PD-CRS and MoCA for dementia classification (89%; *κ* = 0.59, *p* = 0.000).

Significant ceiling effects were found for naming, sustained attention, clock drawing, clock copy, and the cortical subtotal score. However, previous studies have also reported ceiling effects for naming (Pagonabarraga et al., 2008), clock copy (Pagonabarraga et al., 2008; Santangelo et al., 2014; Serrano-Dueñas et al., 2017; Tan et al., 2020), sustained attention (Santangelo et al., 2014), and clock drawing (Santangelo et al., 2014; Tan et al., 2020). This ceiling effect can be explained by the high representation of patients in the early stages in our sample (77%) and short disease duration (median of 4 years) because cognitive impairment can be subtle in early PD. Additionally, the clock copy has been found with a significant ceiling effect in most of the previous studies and is the item with the highest in those studies where additionally there is a high representation of early stages. Also, this item is included in the cortical sub-score which has statistically significant differences between MCI and PD-D but plays no role in differentiating NC from MCI (Pagonabarraga et al., 2008; Tan et al., 2020). This could be explained by the fact that in the early stages, there is almost no compromise in cortical functions; on the contrary, in the late stages of PD or PD-D, both cortical and subcortical functions are compromised as there is a widespread compromise of dopaminergic, cholinergic, and noradrenergic circuits (Weintraub, 2011; Aarsland et al., 2021).

Differences in PD-CRS total score were found according to the Hoehn and Yahr stage as previously mentioned in Serrano’s study (Serrano-Dueñas et al., 2017) as well as in a Chinese cohort (Tan et al., 2020). Likewise, an inverse significant correlation was found between disease duration and PD-CRS. This could reflect the ability of the scale to measure changes in cognition as the disease progresses concomitantly with the worsening of neuropsychological functions (Siciliano et al., 2017; Modestino, 2018). In the Norwegian cohort study, dementia prevalence rose from 27% at baseline to 60% after 12 years (Buter et al., 2008), and in the Sydney cohort, 83% of the patients had dementia after 20 years (Hely et al., 2008). Nonetheless, this correlation is weak which could be due to the fact that cognition status not only relies on disease duration but also depends on the age at the assessment, older age at disease onset, sex, education, overall motor symptoms, and the presence of depression.

Although internal consistency in this study is acceptable (*α* > 0.70) (Thorndike, 1995; Aaronson et al., 2002; Tavakol and Dennick, 2011), results are lower than previously reported in the literature for Spanish (*α* = 0.80–0.85) (Pagonabarraga et al., 2008; Martínez Martín et al., 2009; Fernández-Bobadilla et al., 2017), Chinese (*α* = 0.84)(Tan et al., 2020), Italian (*α* = 0.89)(Santangelo et al., 2014), and Iranian (0.94) (Mahmoudi Asl et al., 2022) populations. Results in this study are comparable to that found in a neighboring population in Ecuador (Guttman’s *λ* 0.821). Guttman’s *λ* as a measure of internal consistency may overestimate the value, while Cronbach’s *α* might underestimate the real internal consistency (Osburn, 2000; Benton, 2015; Green et al., 2016). Differences with previous studies might be caused by specific conditions in the Colombian or Latin American PD populations. Our results are in the same line as the aforementioned studies because removing one of the items does not improve internal consistency and, in turn, could significantly decrease it. Hence, all 9 items are essential to this scale.

MoCA is a recommended scale for Parkinson’s disease cognitive screening (level 1 criteria; Skorvanek et al., 2018) and has been validated in the Colombian population using different cut-off points based on education (Pedraza et al., 2017). A strong correlation was found between MoCA and PD-CRS total scores (Pearson’s *ρ* = 0.71, *p* = 0.000) and for most of the sub-scores. Previous studies have assessed concurrent validity with MoCA showing a significant correlation (Samat et al., 2017; Mahmoudi Asl et al., 2022). Most authors have also assessed concurrent validity finding a significant correlation with other screening tests like the Folstein Mini-Mental State Examination—MMSE (Martínez Martín et al., 2009; Serrano-Dueñas et al., 2017), which has been also validated in the Colombian population for dementia diagnosis, the MDRS-2 (Pagonabarraga et al., 2008; Tan et al., 2020), and the SCOPA-COG (Martínez Martín et al., 2009; Mahmoudi Asl et al., 2022). Only MDRS is a recommended scale for PD cognitive screening, while SCOPA-COG is a “recommended scale with caveats” and MMSE is only suggested because some of their psychometric characteristics have not been found satisfactory (Skorvanek et al., 2018).

Although MoCA test evaluates several cognitive domains, it primarily focuses on attention and executive dysfunctions which characterizes the most common cognitive fronto-subcortical profile in PD (Muslimovic et al., 2005; Pagonabarraga et al., 2008; Kehagia et al., 2013; Gonzalez-Latapi et al., 2021) and early PD, but can miss other cognitive profiles, particularly additional cortical compromises which predict dementia progression (Muslimović et al., 2007; Roheger et al., 2018). PD-CRS is a screening cognitive test that can be easily applied in the neurology consultation and provides useful information by identifying profiles at risk of rapid cognitive deterioration leading to close monitoring.

In this sense, PD-CRS total scores were statistically different among NC, MCI, and PDD subjects (and even between MCI and PDD), suggesting that PD-CRS can accurately differentiate cognition in the three groups. Similar results have been found in previous studies where the total score can also differentiate cognitive status (Pagonabarraga et al., 2008; Fernández de Bobadilla et al., 2013; Fernández-Bobadilla et al., 2017; Mahmoudi Asl et al., 2022). Other investigations pointed out that while subcortical scores differentiate NC from MCI, cortical scores differentiate MCI from PD-D (Pagonabarraga et al., 2008; Rosca and Simu, 2020; Tan et al., 2020); this shows the cognitive progression of the disease wherein advanced states, cortical dysfunction appears (Aarsland et al., 2021). Furthermore, using the PD-CRS cut-off proposed for the Ecuadorian population (Serrano-Dueñas et al., 2017) implies an agreement with MoCA of 89% which suggests that this limit can be acceptable for cognitive screening in the Colombian population until specific cut-offs for our country are defined in future studies.

## Limitations

Even though we included PD patients in early and late stages, 77% of the sample were patients in early stages (H&Y < 2.5) and the median disease duration was 4 years. In these early stages, cognitive impairment can be subtle. Additionally, there is a great percentage of patients with PIGD subtype (84%) which is associated with increased motor and non-motor compromise including faster cognitive decline and lower cognitive-free interval since diagnosis (van der Heeden et al., 2016; Modestino, 2018) and this could influence the results. Mood disorders have an impact on cognition, particularly depression (Hammar, 2009) but also anxiety; however, we only considered previous moderate or severe depression diagnosis as an exclusion criterion, but no screening for this condition was accounted for in the study nor do we applied MDS-UPDRS part I which considers neuropsychiatric symptoms due to time constraints. No follow-up was planned, and no multiple evaluators were considered; therefore; test–retest and inter-rater variability could not be assessed. Comparison and classification of patients in the NC, MCI, and dementia groups were based on another level 1 screening test which is not the gold standard for diagnosis. In the future, we expect to conduct full neuropsychological cognitive and affective evaluations on these patients for overcoming these limitations.

## Future directions

Comparing neuropsychological evaluation (level 2 criteria) with PD-CRS scores is mandatory to establish a specific cut-off point for the Colombian population as it is highly variable among populations (Rosca and Simu, 2020). In addition, the need for age and education adjustment should be explored in further studies. Furthermore, CFA should be run in a subsequent sample for cross-validation.

## Conclusion

PD-CRS has acceptable psychometric properties for the Colombian population and has significant correlation and agreement with a validated scale (MoCA) for PD cognitive evaluation.

## Data availability statement

The raw data supporting the conclusions of this article can be made available by the authors on request, prior approval by the institutional ethics committee. Requests to access these datasets should be directed to beatriz.munoz@fvl.org.co.

## Ethics statement

Informed consent was obtained from all the patients prior to the evaluation as part of a comprehensive PD research protocol within the framework of the LARGE-PD consortium. The research was conducted according to the Declaration of Helsinki and was approved by the local Institutional Review Board in Fundación Valle del Lili: protocol 1245, approval number 150–2018.

## Author contributions

BM-O and JO participated in the design of the study, patient evaluation, interpretation, and revision of data. DA-G and HC-M organized the data, also contributed to its interpretation as well as the writing of the first draft of the manuscript. GP-M contributed to data processing, statistical analysis, and data interpretation and wrote sections of the manuscript. All authors contributed to the article and approved the submitted version.

## Funding

This research project was conducted within the framework of the LARGE-PD consortium with funding from Cleveland Clinic and MJ Fox Foundation.

## Conflict of interest

The authors declare that the research was conducted in the absence of any commercial or financial relationships that could be construed as a potential conflict of interest.

## Publisher’s note

All claims expressed in this article are solely those of the authors and do not necessarily represent those of their affiliated organizations, or those of the publisher, the editors and the reviewers. Any product that may be evaluated in this article, or claim that may be made by its manufacturer, is not guaranteed or endorsed by the publisher.
