# Supplementary material for: Psychometric properties and construct validity of the Parkinson’s Disease-Cognitive Rating Scale (PD-CRS) in Colombia
Source: Front Psychol. 2022 Dec 1;13:1018176. doi: 10.3389/fpsyg.2022.1018176 (PMC9753700; doi:10.3389/fpsyg.2022.1018176)
Supplement: Supplementary file 1 [file Data_Sheet_1.pdf]

INVARIANCE OF THE MODEL STRUCTURE

Results suggest that the factorial loadings and intercept are equivalent in both comparisons, which supports the existence of metric and scalar invariances ($p>0.050$, $\Delta\chi 2/\Delta dof <3$). Nevertheless, there was no support for strict invariance as the residual differences were indeed different.

Supplementary table 1. Invariance of the model structure by motor subtype (PIGD n=84 vs. tremorous/undetermined n=16)

Invariance	Δχ2	P value	Δdof	Δχ2 / Δdof	ΔRMSEA	ΔCFI	ΔTLI	ΔSRMR
Metric	8.678	0.277	7	1.239	0.003	-0.018	-0.01	0.017
Scalar	9.179	0.240	7	1.311	0.001	-0.013	-0.003	0.005
Strict	22.841	0.007	9	2.498	0.024	-0.105	-0.088	0.076

Supplementary table 2. Invariance of the model structure by cognitive impairment (MCI/dementia on MoCA n=41 vs. without impairment n=59)

Invariance	Δχ2	P value	Δdof	Δχ2 / Δdof	ΔRMSEA	ΔCFI	ΔTLI	ΔSRMR
Metric	12.667	0.080	7	1.809	0.032	-0.080	-0.094	0.023
Scalar	7.7652	0.354	7	1.109	-0.003	-0.001	0.012	0.007
Strict	25.248	0.003	9	2.805	0.020	-0.106	-0.088	0.049
